# New Lidocaine-Based Pharmaceutical Cocrystals: Preparation, Characterization, and Influence of the Racemic vs. Enantiopure Coformer on the Physico-Chemical Properties

**DOI:** 10.3390/pharmaceutics15041102

**Published:** 2023-03-29

**Authors:** Panpan Ma, Balthazar Toussaint, Enrica Angela Roberti, Noémie Scornet, Axel Santos Silva, Luis Castillo Henríquez, Monique Cadasse, Philippe Négrier, Stéphane Massip, Hanh Dufat, Karim Hammad, Cecilia Baraldi, Maria Cristina Gamberini, Cyrille Richard, Stéphane Veesler, Philippe Espeau, Tu Lee, Yohann Corvis

**Affiliations:** 1 CNRS, INSERM, UTCBS, Chemical and Biological Technologies for Health Group, Université Paris Cité, F-75006 Paris, France; 2 Département Recherche et Développement Pharmaceutique (DRDP), Agence générale des Équipements et Produits de Santé, AP-HP, F-75005 Paris, France; 3 Département Physico-Chimie du Médicament, Unité Pédagogique de Chimie Analytique, Physique et Toxicologie, Faculté de Santé, Université Paris Cité, 4 Avenue de l’Observatoire, F-75006 Paris, France; 4 Laboratoire Ondes et Matière d’Aquitaine, Université de Bordeaux, UMR 5798, F-33400 Talence, France; 5 CNRS, INSERM, IECB, Université de Bordeaux, UAR 3033, F-33600 Pessac, France; 6 CiTCoM, Université Paris Cité, UMR CNRS 8038, F-75006 Paris, France; 7 Department of Life Sciences, University of Modena and Reggio Emilia, 41125 Modena, Italy; 8 CNRS, Aix-Marseille Université, CINaM (Centre Interdisciplinaire de Nanosciences de Marseille), Campus de Luminy, CEDEX 09, F-13288 Marseille, France; 9 Department of Chemical and Materials Engineering, National Central University, Taoyuan 320317, Taiwan

**Keywords:** solid state, crystal engineering, lidocaine, dl-menthol, physico-chemical compatibility, thermodynamic stability, cocrystallization, dissolution kinetics, solubility enhancement

## Abstract

This study describes the preparation, characterization, and influence of the enantiopure vs. racemic coformer on the physico-chemical properties of a pharmaceutical cocrystal. For that purpose, two new 1:1 cocrystals, namely lidocaine:dl-menthol and lidocaine:d-menthol, were prepared. The menthol racemate-based cocrystal was evaluated by means of X-ray diffraction, infrared spectroscopy, Raman, thermal analysis, and solubility experiments. The results were exhaustively compared with the first menthol-based pharmaceutical cocrystal, i.e., lidocaine:l-menthol, discovered in our group 12 years ago. Furthermore, the stable lidocaine/dl-menthol phase diagram has been screened, thoroughly evaluated, and compared to the enantiopure phase diagram. Thus, it has been proven that the racemic vs. enantiopure coformer leads to increased solubility and improved dissolution of lidocaine due to the low stable form induced by menthol molecular disorder in the lidocaine:dl-menthol cocrystal. To date, the 1:1 lidocaine:dl-menthol cocrystal is the third menthol-based pharmaceutical cocrystal, after the 1:1 lidocaine:l-menthol and the 1:2 lopinavir:l-menthol cocrystals reported in 2010 and 2022, respectively. Overall, this study shows promising potential for designing new materials with both improved characteristics and functional properties in the fields of pharmaceutical sciences and crystal engineering.

## 1. Introduction

Solubility issues of active pharmaceutical ingredients (APIs) demand a thorough approach to make the compounds suitable for formulation to enhance their therapeutic efficacy. Large numbers of APIs with high permeability are conditioned by their poor water-solubility (i.e., class II of the Biopharmaceutics Classification System, BCS) [1,2]. Cocrystals have been developed for nearly three decades to address this issue [3,4,5], and to date, eight cocrystal-based pharmaceutical products have been identified on the market [6,7]]. Cocrystals are non-covalent associated substances containing at least two different molecular components: An API and a cocrystal former, namely coformer [8,9,10,11,12]. From a pharmaceutical point of view, the coformers can be another API(s) and/or excipient(s). In a single homogenous crystal phase, different components among cocrystals have a definite structure with a stoichiometric ratio. Furthermore, cocrystals are stabilized via different molecular interactions: Van der Waals forces, hydrogen bonds, and π–π stacking. Remarkably, the coformer(s) and the API(s) can interact non-ionically, which distinguishes them from salts [12].

Cocrystallization improves the solubility of poorly water-soluble drugs as these usually do not have the ionic functional groups to facilitate salt formation. The solubility enhancement is due to the reduction in the solvation barrier of the entire cocrystal system owing to the coformer presence and can be adjusted to a certain extent by altering the pH of the solution, coformer concentration, or solubilizing agent concentration [10,13]. Comprehensive cocrystal formations can be found in numerous reports [14,15]. Major methods of cocrystal preparation are described in the literature such as solvent evaporation, solid-state grinding, liquid-assisted grinding, slurrying, anti-solvent cocrystallization, supercritical fluids, sonocrystallization, spray drying, resonant acoustic mixing, direct cocrystal assembly [16], and twin-screw extrusion techniques [17,18,19]. Among them, solvent-drop grinding has been shown to be an efficient method for the effective detection of the majority of cocrystals [20,21] and polymorphism control of the cocrystallization [22]. Therefore, cocrystallization techniques allow bypassing the dissolution rate as the limiting step, and thus, improve the overall biopharmaceutical properties of the API formulated as cocrystals [23]. Apart from solubility, cocrystallization can also improve other physicochemical properties of the drug product, including mechanical, chemical stability, flowability, compressibility, hygroscopicity [24], tableting properties [20], and also in vivo therapeutic activity [25]. As an illustration, the drug melting point can be modified via cocrystal formation [26]. In addition, cocrystals are preferred to hydrates or solvates because of higher stability during storage, where the chosen coformer plays a fundamental role in achieving that [27].

Since 1844 when the first cocrystal, quinone:hydroquinone, was discovered [28], various applications of pharmaceutical cocrystals have been proposed for enhanced therapeutic effect [29]. Among them, lidocaine, well-known as an amide-type anesthetic compound, is used as standard practice for topical drug delivery before venipuncture [30] or minor surgeries [31], during endoscopy procedures [32,33], and even after laser skin treatments [34]. Lidocaine can also be parenterally administered for invasive clinical procedures such as colonoscopy [33,35] or awake tracheal intubation [36]. l-menthol [37,38], a transdermal enhancer agent for API [39], was chosen as a generally recognized as safe (GRAS) pharmaceutical excipient for cocrystal preparations using topical anesthesia [40]. Furthermore, l-menthol can also be considered an active ingredient for cough medicine [41], since it presents some cooling and local anesthetic properties [42]. Indeed, menthol as a hydrophobic molecule (log P = 3.4) can interact in vivo with biological membranes. Since the pharmacological action of active ingredients is governed by intermolecular interactions with the biological environment [43], the menthol absolute configuration (levogyre, dextrogyre, or racemate) in a final product is crucial to obtaining its proper therapeutic activity.

Twelve years ago, the first menthol-based cocrystal, namely the lidocaine:l-menthol cocrystal (Lido: lM), was discovered in our group during research experiments on therapeutic deep eutectic preparation for improved anesthetic cream formulations [44]. The latter cocrystal was obtained by the thaw–freeze technique from the API/coformer molten state. In order to obtain some insights into (i) the preparation generalization, (ii) the interaction forces governing the cocrystal formations, and (iii) the enantiopure/racemic dependency on the cocrystal synthesis, two other menthol entities as coformer were tested for cocrystallization. Interestingly, two new cocrystals, namely lidocaine:d-menthol (Lido:dM) and lidocaine:dl-menthol (Lido:dlM) were designed and characterized by X-ray diffraction, infrared spectroscopy, Raman, thermal analysis, and solubility measurements. The results were also compared with new physicochemical data on the Lido:lM cocrystal. Altogether, the cumulative data highlight Lido:dlM cocrystal as the most convenient for potential pharmaceutical applications. Indeed, six pharmaceutical specialties with the lidocaine/l-menthol association are currently sold on the French market. These medicines are indicated for the short-term treatment of mild sore throat without fever, owing to their anesthetic, antiseptic, and antibacterial properties. Two out of these six specialties are prepared with a lidocaine-free base, while the others are composed of hydrochloride lidocaine salt. In most cases, a free base of a given drug can present the advantage to improve the sustained release of the API [29,45].

Furthermore, it has been recently proven that the second menthol-based pharmaceutical cocrystal designed to date, i.e., the lopinavir:l-menthol 1:2, promotes the intestinal absorption of the HIV-1 protease inhibitor [46]. Consequently, it can be proposed that the cocrystals prepared from menthol and a free base API may enhance both the permeability and solubility of the latter. Interestingly, to date, no preparation with the lidocaine/racemic menthol association has been marketed. LidoPatch^®^ (J.A.R. Laboratories, Lake Forest, IL, USA), composed of 3.6% lidocaine hydrochloride and 1.25% menthol, is marketed as an over-the-counter (OTC) drug in several countries. However, the menthol physico-chemical characteristic is not specified, while a lidocaine salt is used. Nevertheless, a study published in 2017 has demonstrated better safety, therapeutic efficacy against back pain and arthritis, and impact on the patient’s (*n* = 77) quality of life for LidoPatch^®^ compared to a prescription patch formulation with 5% lidocaine [47]. Therefore, the new lidocaine/menthol-racemate association proposed in the present study presents some biopharmaceutical advantages for the improvement of local delivery of lidocaine. Alternatively, some menthol-based cocrystals with non-therapeutic properties have been reported in the literature, e.g., for the food industry [24], or engineering new material survey [48,49].

## 2. Materials and Methods

### 2.1. Materials

Lidocaine (purity: 97.5%; CAS 137-58-6), l-menthol (purity: 99.7%; CAS 2216-51-5) were purchased from Acros Organics (Morris Plains, NJ, USA), d-menthol (purity: 99%; CAS 15356-70-4) was obtained from Janssen Chimica (Geel, Belgium), and dl-menthol (purity: >98%; CAS 89-78-1) was provided by Alfa Aesar (Karlsruhe, Germany). No further purification steps were required since the cocrystals are directly obtained from the intermolecular interactions of the pure compounds. For the buffer preparation, Na_2_HPO_4_ and KH_2_PO_4_ were purchased from Acros Organics, and ultrapure water was prepared via filtration of distilled water through 2 ion-exchange membranes (resistivity = 18.2 MΩ·cm at 25 °C), with a final filtration through a 0.2 µm membrane (Milli-Q^®^, Merck, Darmstadt, Germany).

### 2.2. Cocrystal Preparation and Storage

The stoichiometric compounds were achieved by melting a mixture of lidocaine with d-menthol (obtaining Lido:dM cocrystal) and dl-menthol (obtaining Lido:dlM cocrystal) separately in equimolar proportion at 90 °C in glass flasks. Then, the flasks were quenched first at −80 °C for 10 min to obtain a glassy phase and finally heated from −80 °C to room temperature (22–26 °C). Spontaneously, crystals of the 1:1 compound were formed by nucleation during the warming of the sample. The flasks were sealed in order to avoid d-menthol or dl-menthol evaporation during the heating and cooling processes. The cocrystals were stored in their solid state in sealed flasks at 5 °C for at least 10 years without any physical or chemical modification, confirmed by the melting point assessment.

### 2.3. Single Cocrystal Engineering

Cocrystal powder was placed on a glass slide positioned under a microscope at room temperature. Then, a small amount of l- or dl-menthol powder was added to the cocrystal powder, leading to the melting of the overall system due to the eutectic phenomenon. Due to the relatively high vapor pressure of both racemic and enantiopure menthol compounds [50,51,52], evaporation of the excess molten menthol was achieved after some minutes of the mixture exposure to air at room temperature, inducing the monocrystal formation of the l- or dl-menthol-based cocrystal. The single cocrystals were stored in their solid state in sealed flasks at room temperature, avoiding exposure to light, for at least 10 years without any physical or chemical modification, confirmed by single crystal X-ray diffraction experiments.

### 2.4. Physical Mixtures Preparation for the Establishment of the Stable Lidocaine/dl-Menthol Phase Diagram

The physical mixtures between dl-menthol and Lido:dlM and between lidocaine and Lido:dlM, were achieved at 4 °C in a cold room to avoid any melting during mixing. For that purpose, the pure monocomponent compounds were weighed separately at room temperature in a weighing container of known mass and then transferred to the cold room to be combined by grinding via a mortar and a pestle. After the transfer of each monocomponent powder, the weighing containers were then weighed at room temperature in order to calculate the exact molar ratio for each mixture sample.

### 2.5. Buffer Preparation

The phosphate buffer (50 mM, pH = 7.2) used for the dissolution experiments was prepared from the phosphate disodium and monopotassium salts (Na_2_HPO_4_ and KH_2_PO_4_, respectively; p*K*_a_ = 7.2). The phosphate salts were dissolved in ultrapure water; salt concentrations: 30.7 and 19.3 mM for Na_2_HPO_4_ and KH_2_PO_4_, respectively, and then a few microliters of NaOH 1 M were added to the solution in order to adjust the final pH to 7.2. Finally, the buffer solution was filtrated through a 0.45 µm cellulose mixed esters membrane (Carl Roth, Karlsruhe, Germany) prior to its use.

### 2.6. X-ray Diffraction

X-ray Powder Diffraction patterns were collected with a vertically mounted INEL cylindrical position-sensitive detector (CPS-120) using the Debye–Scherrer geometry and transmission mode providing an angular step of 0.029° (2θ) between 4 and 120°. External calibration using NAC (Na_2_Ca_2_Al_2_F_14_) cubic phase mixed with silver behenate (AgC_22_H_43_O_2_), the last one suitable for low-angle was performed by means of cubic spline fittings in order to correct the angular linearity deviation of the detector according to the recommended procedure [53,54]. Monochromatic Cu-Kα_1_ (λ = 1.540562 Å) radiation was selected through an asymmetrically focusing incident-beam curved quartz monochromator. The generator power was set to 1.0 kW (40 kV; 25 mA). The samples were introduced in 0.5 mm diameter Lindemann capillaries which rotate perpendicularly to the X-ray beam during the experiments to improve the averaging of the crystallite. The indexing of the X-ray patterns, structure solutions, and Pawley and Rietveld refinements was performed using the Materials Studio Program version 5.5 (BIOVIA, San Diego, CA, USA) [55]. Molecular energy minimization with the Forcite module using the Dreiding forcefield available in the Materials Studio Program software has been used to build up rigid body molecules. To solve the structure, rigid molecules of l-menthol and lidocaine were placed in a randomly oriented general position, and through the Powder Solve using the Monte-Carlo approach, both the position and orientation of molecules within the unit lattice were found. After refinement, the reliability factors obtained were not satisfactory. Indeed, although the space group is centered, the monocrystal data showed that the l-menthol molecule is disordered with two levogyre menthol molecules in the same place. The second molecule was obtained from the first one after two 180-degree rotations (Table S1). The first one was carried out in the mean plane of the molecule and the second in a plane perpendicular to the mean plane of the molecule. This allows the oxygen atom to return to a good position to have an H bond with lidocaine. d-menthol molecules were obtained through the symmetry center of the space group. The ultimate disordered structural solution was obtained employing Rietveld refinement, in which the position and orientation of the molecules, within the rigid-body constraint, with a single overall isotropic displacement parameter, were refined. The obtained R factors were Rwp = 5.49% and Rp = 3.85%.

X-ray analyses of single cocrystals were carried out on an FR-X Rigaku diffractometer with a rotating anode at monochromatic Cu-Kα radiation (λ = 1.54184 Å) and a Pixel Hybrid detector HyPix 6000. Data collection and reduction were performed with CrysAlisPro [56]. The structure was solved by direct methods and refined using the Shelx 2014 suite of programs [57] in the integrated WinGX system (University of Glasgow, Glasgow, UK) [58]. The positions of the H atoms were deduced from the coordinates of the non-H atoms and confirmed by Fourier synthesis. The non-H atoms were refined with anisotropic temperature parameters. H atoms were included for structure factor calculations but not refined. The program Mercury CSD version 2.0 (University of Cambridge, Cambridge, UK) [59] was used for analysis and drawing figures.

### 2.7. Spectroscopy Experiments

Infrared and Raman experiments were performed with the samples in their solid states. The pure single components were used without any treatment. The cocrystals were scratched from their compact solid organization. Fourier-transform IR (FTIR) data were acquired on a FT-IR spectrometer using attenuated total reflectance, UATR 1 Bounce model (PerkinElmer SAS, Courtaboeuf, France) in absorbance mode with 4 cm^−1^ resolutions and 50 scans. The scans were conducted at room temperature in the wavenumbers range of 4000 to 650 cm^−1^ for each sample placed on a zinc selenide (ZnSe) surface and in contact with a stainless-steel tip. Spectrum 65 L125100I software (PerkinElmer SAS, Courtaboeuf, France) was used to collect and analyze the spectra data. The Raman analyses were conducted with a confocal micro-Raman LabRAM HR evolution (Horiba Jobin Yvon, Edison, NJ, United States), equipped with a red He-Ne laser at 632.8 nm (1 μm-size spot), notch filters to eliminate the exciter, detector CCD front illuminated open electrode multi-pin phased, with 1024 × 256 × 16 pixels, cooled by a Peltier system. Spectra were recorded in backscattering after focalization in several positions within a small area of the sample (*ca.* 100 µm × 100 µm). The maximum laser power employed was 20 mW and the recording time for a good signal-to-noise ratio was 5 s for 10 accumulations. Furthermore, GRAMS/AI 7.02 was used for the spectra elaboration.

### 2.8. Thermal Analysis Experiments

After a calibration procedure using high-purity indium (99.99%, T_fus_ = 156.6 ± 0.3 °C, Δ_fus_H = 28.45 ± 0.60 J·g^−1^) and zinc (99.99%, T_fus_ = 419.6 ± 0.7 °C, Δ_fus_H = 107.5 ± 3.2 J·g^−1^) reference specimens, temperatures and enthalpies of fusion were settled using a differential scanning calorimeter (DSC 822e from Mettler Toledo, Greifensee, Switzerland). The samples (mass between 1 and 5 mg) were introduced in aluminum pans, hermetically sealed with a Mettler Toledo press, and weighed to obtain the exact amount of sample for each pan. The heating program was managed at 5 K·min^−1^ under a dry nitrogen gas atmosphere (flow rate: 50 mL·min^−1^). Thermogravimetric analyses (TGA) were performed using a TGA/DSC 1 from Mettler Toledo, sensitive to 1 µg. Samples of approximately 5 mg were analyzed at a heating rate of 10 K·min^−1^ under nitrogen gas (flow rate: 50 mL·min^−1^) in pin-holed aluminum pans obtained with a stainless-steel tip. The mass of the analyzed samples was recorded using TA universal analysis software. The thermal analysis experiments were conducted in triplicate and were evaluated with the Stare 16.30 software (Mettler Toledo, Greifensee, Switzerland).

### 2.9. Nuclear Magnetic Resonance Experiments

Proton nuclear magnetic resonance (^1^H NMR) spectra were recorded at 400 MHz on a Brüker AVANCE III, using either D_2_O (deuterated water) or CDCl_3_ (deuterated chloroform) as a solvent, and have been analyzed using TopSpin 4.1.3 software. All samples were prepared at 8.3 mg·mL^−1^ and dissolved at room temperature for 24 h and 10 min in D_2_O and CDCl_3_, respectively. To obtain more insights into the menthol/lidocaine interactions in CDCl_3_, 2D Nuclear Overhauser Effect SpectroscopY (NOESY) spectra were recorded with the same instrument. For this purpose, a lidocaine/dl-menthol mixture was prepared in CDCl_3_ with the same molar ratio as the Lido:dlM cocrystal dissolved in the latter solvent.

### 2.10. Density Measurement

An electronic density meter, namely DMA 5000 (Anton Paar, Graz, Austria) was used to obtain the solubility data for lidocaine, Lido:lM, and Lido:dlM. The device is equipped with a borosilicate U-shape tube (volume thereabouts 1 mL) calibrated beforehand with dry air and ultrapure Milli-Q^®^ water. Its working principle is to maintain in oscillation the U-shape tube filled with a given sample knowing that the measurement of the resulting period is proportional to the volumetric mass of the sample, allowing to determine also the density. For sample preparation, an excess amount of cocrystal was introduced into a 10 mL volumetric flask filled with the phosphate buffer solution (pH = 7.2). The resulting suspension homogenized at room temperature was agitated by a magnetic stirrer for at least 12 h. Each suspension was then filtered through a 0.45 µm cellulose mixed esters membrane (Carl Roth, Karlsruhe, Germany), and afterward, the filtered saturated solution was introduced into the density meter capillary already set at 25 °C. After 30 s of equilibrium, the measurement of the density was launched. The results were treated as a function of the theoretical lidocaine concentration.

### 2.11. Kinetics of Dissolution

For the kinetics of dissolution investigations, an experimental setup developed by ANACRISMAT (Marseille, France) was used [60]. The device is composed of a Nikon Diaphot inverted optical microscope (Nikon, Kawasaki, Japan) and a mono-well crystallization cell MW1(ANACRISMAT, Marseille, France) used in conjunction with a CCD camera (Imaging Source DFK 31BF03, Bremen, Germany). The focus of the microscope was adjusted on the bottom of a 3 mL flask connected to a Peltier temperature control unit (25 ± 0.1 °C). This set-up has been conceived to fit an optical holding cell in which a powder sample can be observed with or without any solvent. For the present study, a few milligrams of pure lidocaine, pure dl-menthol, Lido:dlM, or Lido:lM were introduced in the 3 mL flask. An optical image was then taken for each system. Finally, the timer was triggered when 2 mL of phosphate buffer was introduced for each system, the flask was sealed with a glass stopper to avoid evaporation, and optical images were taken at different time points. The dissolution was therefore followed under quiescent conditions.

## 3. Results and Discussion

### 3.1. Design and Structure of Two New Cocrystals

**Figure 1:**
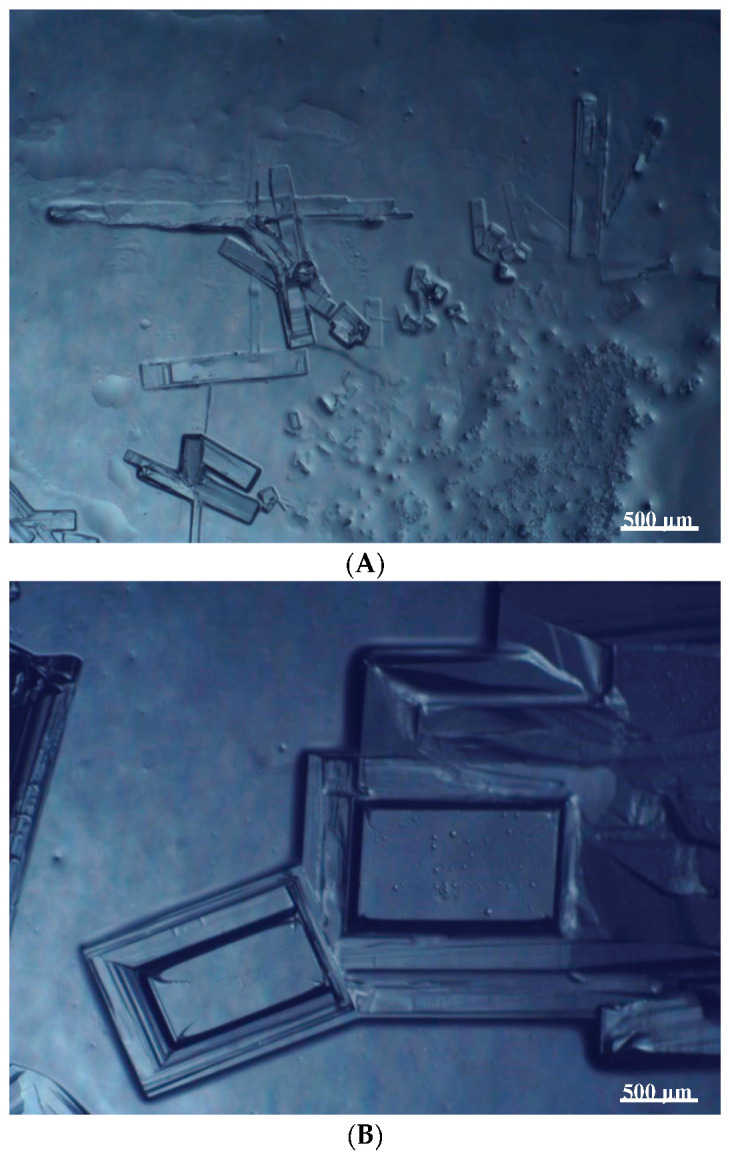
Optical microscopy image of the lidocaine:l-menthol (**A**); and lidocaine:dl-menthol (**B**) cocrystals.

**Figure 2:**
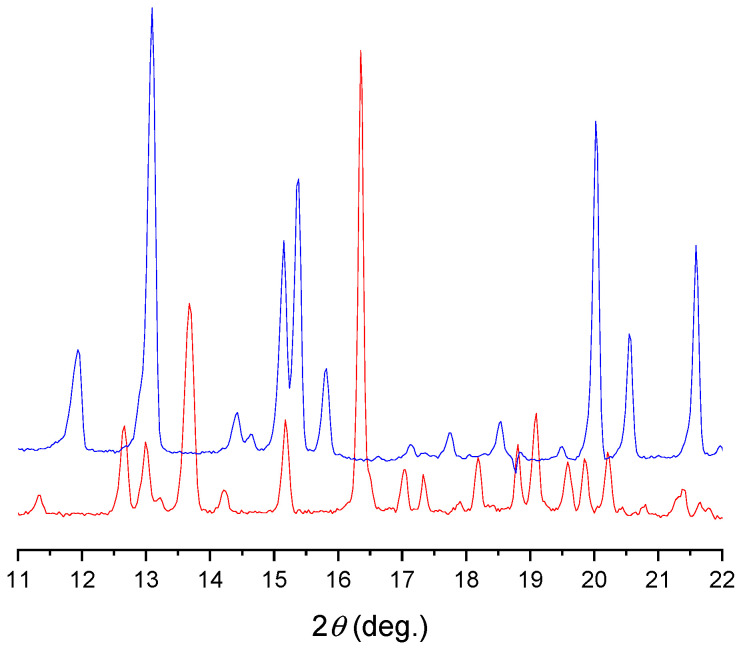
X-ray patterns obtained for the lidocaine:l-menthol cocrystal (upper spectrum), and the lidocaine:dl-menthol cocrystal (lower spectrum) at 22 °C.

**Figure 3:**
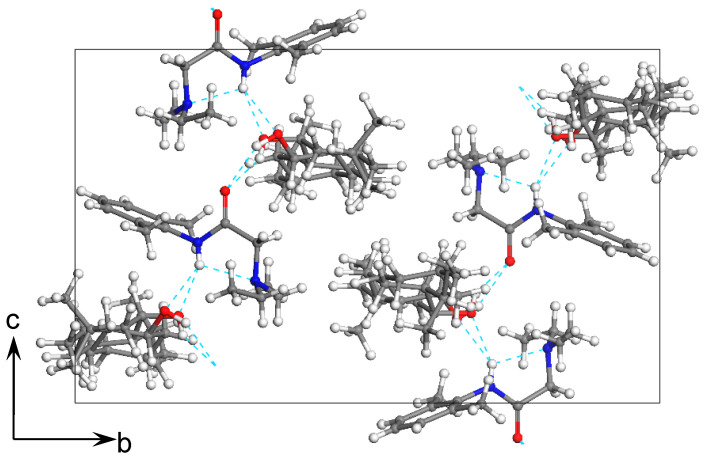
Crystal structure of the lidocaine:dl-menthol cocrystal. Hydrogen bonds are indicated by dashed cyan lines.

To extend our findings concerning the lidocaine-l-menthol cocrystallization in the context of racemate vs. enantiomer influence, new formulations with racemic menthol and d-menthol were tested by adapting the same procedure of preparation, respectively. Firstly, the morphology of the racemic and enantiopure cocrystals was observed under an optical microscope (Figure 1). The grown crystals from the lidocaine/l-menthol stable system present a thin rectangular-like shape, while those obtained from the lidocaine/dl-menthol present a larger size with a thicker tabular habit. X-ray patterns of the corresponding powders, recorded at 296 K, highlighted the existence of a 1:1 cocrystal obtained between lidocaine and dl-menthol, namely Lido:dlM (Figure 2). Noteworthily, the X-ray pattern for the Lido:dM overlaps that of Lido:lM, suggesting the formation of a third lidocaine-menthol cocrystal since the two latter systems differ only in the inversion center (results not shown). The Rietveld refinement from the Lido:dlM powder diffraction pattern allowed us to obtain the crystal structure of the new racemic cocrystal formed with menthol racemate and lidocaine (Figure 3). This cocrystal crystalizes in a P 2_1_/c monoclinic space group (Table 1), while the Lido:lM one crystalizes in a P2_1_2_1_2_1_ space group [44]. These results were confirmed by the X-ray diffraction performed on a single Lido:dlM cocrystal at 100 and 233 K. Cooling of the crystal did not induce any menthol ordering at 100 K. Thus, all results highlighted disordered menthol molecules for the menthol racemate-based cocrystal (Table S1). Each site in the unit cell is occupied by only one enantiopure menthol, i.e., l- or d-. In addition, the residual electron density performed with the Lido:dlM cocrystal shows a possible third disordered molecule on this site. However, we failed in positioning it, either l- or d-menthol. Indeed, all refinements of a third disordered menthol in the asymmetric unit led to unstable results. Interestingly, we have previously proven that pure l-, d- or dl-menthol crystalizes with three independent molecules [37] whereas the lidocaine:menthol cocrystals crystalize with a single menthol molecule in the asymmetric unit. The X-ray diffraction data were indeed evaluated regarding the enantiopure menthol-based cocrystal that has been previously reported [44]. In the unit cell of both 1:1 cocrystals, a menthol molecule interacts with two different lidocaine molecules via hydrogen bonding. The crystallographic data of powder (CCDC reference number 2232113) and single crystal structures of the Lido:dlM cocrystal at 100 and 233 K (CCDC reference numbers 2,231,851 and 2248363, respectively) have been deposited in the Cambridge Crystallographic Data Centre (CCDC). The differences in torsion angles between the enantiopure and the racemic cocrystals are presented in the Table S1 of the Supplementary Materials. As far as the H-bonding is concerned, it has been previously demonstrated that the formation of two new intermolecular O-H···O and N-H···O types between l-menthol and lidocaine molecules allows stabilizing the Lido:lM cocrystal [44]. Comparing the intermolecular H-bonding network of the enantiopure- vs. the menthol racemate-based cocrystals, the latter cocrystal presents shorter lengths and smaller angles (Table S2), and more importantly, two disordered menthol molecules in a unit cell (Tables S1 and S2), suggesting a less dense crystal structure. This statement is confirmed by the low density of the menthol racemate-based cocrystals: 1.040 and 1.025 g·cm^−3^ for the Lido:lM [44] and Lido:dlM (Table 1) cocrystals, respectively.

### 3.2. Physico-Chemical Properties of the l-, d- and dl-Menthol-Based Cocrystals

Since the lidocaine:l-menthol 1:1 cocrystal has been proposed for anesthetic drug formulations in their solid form [44], it is interesting to compare its physico-chemical properties to those of the lidocaine:dl-menthol 1:1 cocrystal. Interestingly, the latter presents a melting point decrease of 7 °C compared to the enantiopure cocrystal (Table 2). This can be explained by the fact that racemic menthol presents a depletion of 9 °C by comparison with enantiopure menthol [37]. Surprisingly, while the heats of fusion of the racemic and enantiopure menthol compounds are identical [37], the menthol racemate-based cocrystal one is 25% lower than that of the enantiopure cocrystal. All these findings suggest that Lido:dlM is the least thermodynamically stable cocrystal among the three lidocaine-based ones from the present study, confirming the discussion above regarding the comparison of the two cocrystal structure. Consequently, the pharmaceutical applications of Lido:dlM may differ from that of Lido:lM. Furthermore, the higher heats of fusion of the cocrystals compared to that of the pure compounds show the thermodynamic driving force for forming both cocrystals from the single components [61].

**Figure 4:**
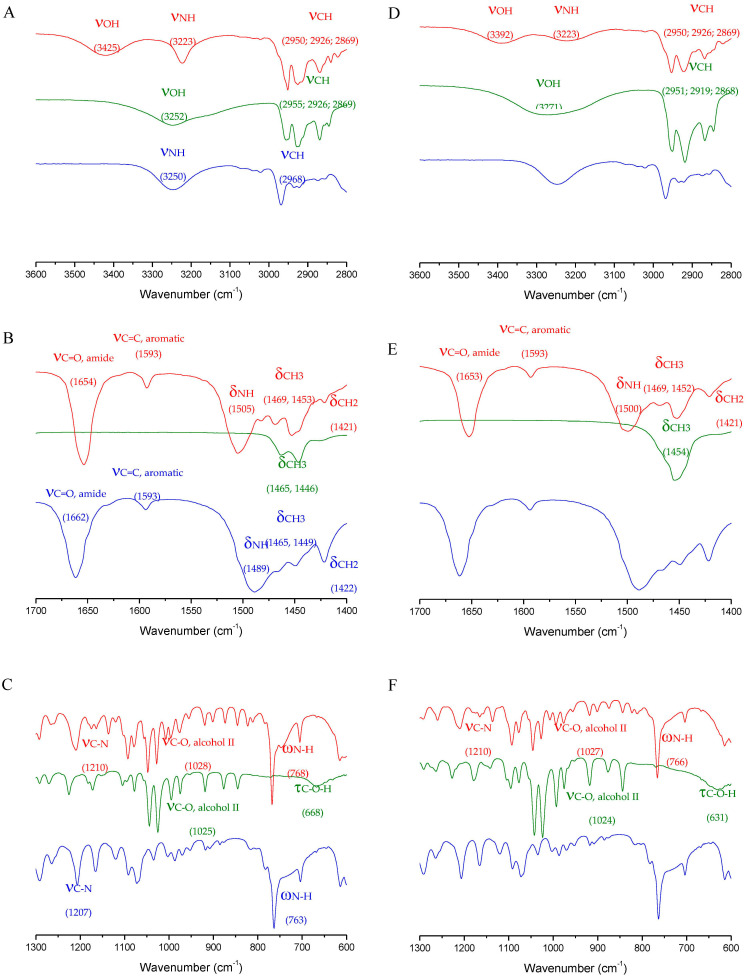
Infrared spectra obtained for the l- (**left column**, red curves) and dl-menthol-based (**right column**, red curves) cocrystals. Infrared spectra for lidocaine (blue curves) and menthol (green curves, i.e., l- and dl-menthol in the left and right columns, respectively) have been presented for comparison. The curves have been shifted for clarity. (**A**,**D**), (**B**,**E**), and (**C**,**F**): spectra in the 3600–2800, 1700–1400, and 1300–600 cm^−1^ wavenumber regions, respectively. The most important peak attributions are proposed on the curves with, the related wavenumber proposed in brackets in cm^−1^.

The interpretations of the X-ray experiments have also been confirmed by infrared (IR) and Raman spectroscopic experiments (Figure 4 and Table 3, respectively).

Indeed, infrared spectroscopy was performed to confirm and validate the formation of cocrystals by the weak bonding interactions between menthol and lidocaine, illustrating the new hydrogen bond linkage and alterations in bond strength for both racemic and enantiopure cocrystals. The amide moiety of lidocaine interacts with the hydroxyl moiety of the menthol since there is a decrease in the ν_C=O_ from 1662 cm^−1^ for the lidocaine spectrum to 1654 cm^−1^ for both l- and dl-menthol-based cocrystals. As can be seen in Figure 4B,E, they have a similar overall behavior since they present the same chemical formula. However, the racemic form (Figure 4C) shows differences in some areas throughout the spectrum. Some of these differences are shifted towards the right or left due to the weakening or strengthening of bonds. Firstly, in the l- and dl-menthol-based cocrystals, a small decrease in strength from the aromatic coming from the menthol can be noticed as the two signals at 1027 and 1048 cm^−1^ were slightly shifted to lower wavenumbers (1024 and 1044 cm^−1^). However, for the Lido:dlM cocrystal, the peak positions are in agreement with the dl-menthol spectrum. Furthermore, there is a double aromatic stretched peak coming from the lidocaine displayed at 1072 and 1092 cm^−1^ with the first peak showing a higher intensity than the second one. For the cocrystal, the first peak is at 1079 cm^−1^ and the second one holds the same position at 1092 cm^−1^; however, the second peak has a higher intensity than the first one in the dextrogyre- and levogyre-based lidocaine:menthol cocrystals. It is worth mentioning that the same peaks can also be found in the menthol molecules with a much lower intensity and slightly stronger bond according to the wavenumber. On the other hand, racemic menthol has a much more pronounced peak around that region being mimicked by the dl-menthol-based cocrystal, where the peak at 1092 cm^−1^ is once again the strongest and also its intensity is slightly higher than that of menthol.

Another interesting visual aspect comes from the hydroxyl and amine groups, in the dl- and l-menthol-based cocrystals, the two peaks can be seen at 1173 and 1163 cm^−1^ corresponding to the secondary OH moiety stretching, which is close to the lidocaine amine signal at 1165 cm^−1^, whereas the dl-menthol-based cocrystal exhibits only one broad peak at 1165 cm^−1^ from amine but the alcohol signal is not as intense as the latter. Moreover, in the FTIR spectrum region that corresponds to the hydrogen bond formation, the two cocrystals revealed two peaks: One related to amine and the other related to alcohol hydrogen bonds. In the corresponding pure menthol and lidocaine, ν_OH_ and ν_NH_, respectively, impacted by the hydrogen bond network, are at ~3250 cm^−1^. Once the cocrystals are formed, two long wave-shaped peaks, one at 3223 cm^−1^ corresponding to amine and another at 3425 cm^−1^ corresponding to the OH, agree with our previously published results [44]. However, the Lido:dlM cocrystal does not share the same wavenumber position nor the same shape as the enantiopure cocrystal regarding the ν_OH_ and the ν_NH_ signals, respectively. Indeed, while the wavenumber of the ν_OH_ signal is lower for the racemic cocrystals than for the enantiopure one (3392 vs. 3425 cm^−1^), its ν_NH_ signal is broader. The first difference confirms the X-ray diffraction results revealing that the length of the intermolecular O−H···O interaction is shorter for the Lido:dlM cocrystal (Table S2). Interestingly, the broad v_NC_ signal can be explained by the molecular disorder also highlighted in the discussion of the X-ray diffraction experiments, i.e., the torsion angles (Table S1) and the lower density (Table 1). Although the gain of stability of cocrystals with respect to their pure components is generally difficult to assess [62], one can confirm from the infrared data that the Lido:dlM cocrystal is less thermodynamically stable than the enantiopure one, validating the thermal analysis results. Raman spectroscopic analysis (Table 2) also corroborated these findings.

Although the presence of polar groups such as -NH and -OH bands are less evident, if not absent in Raman, the micro-Raman technique allowed to distinguish the racemic menthol from the enantiopure menthol-based cocrystal. Pure l-menthol and dl-menthol, on the other hand, have similar spectra. In particular, the strongest bands attributed to the CH stretching bonds are at 2964–2931–2859 cm^−1^ for the l-menthol, while they are shifted to 2958–2926–2867 cm^−1^ for the racemate. These differences are also emphasized in the respective cocrystals with lidocaine. Furthermore, the bands at 876 and 809 cm^−1^ are stronger for l-menthol, while for the racemate the peak at 767 cm^−1^ is highlighted as more intense. l-menthol still exhibits bands at 546/540, 404, 291 as opposed to the strong band at 553 cm^−1^ and at 408, 289 cm^−1^ for the racemate. Furthermore, comparing the Raman spectra of pure lidocaine and menthol with their respective cocrystals, confirms the involvement of the amide group in a hydrogen bond, as already found in infrared. Indeed, an intense band at 1651 cm^−1^ appears in all the cocrystals. This band is associated with HCN scissoring deformation, which falls at 1591 cm^−1^ for pure lidocaine and upshifts due to hydrogen bonding. In the lidocaine hydrochloride salt, this band is also present [63]. The appearance of a band at 543 cm^−1^ in the Lido:lM cocrystal and at 546 cm^−1^ in the Lido:dlM cocrystal is associated with the NCO twist of the amide conditioned by the hydrogen bond network. The Raman spectra are displayed in the Supplementary Materials (Figure S1).

### 3.3. Screening of the Solid–Liquid Equilibria for the dl-Menthol-Based Cocrystal

**Figure 5:**
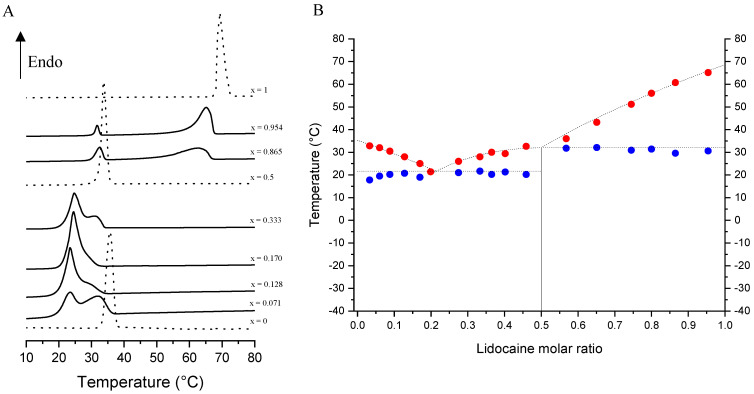
(**A**) Differential scanning calorimetry thermograms obtained for pure compounds, namely dl-menthol (···, bottom curve), Lido:dlM 1:1 stoichiometric compound (···, middle curve), and lidocaine (···, top curve), for dl-menthol/Lido:dlM mixtures (–, the 4 bottom curves), and for Lido:dlM/lidocaine mixtures (–, the 2 top curves). The lidocaine molar ratio, x, is indicated for each sample. Endothermic transformations give signals which point up. (**B**) Related stable Lido:dlM phase diagram. The experimental data (full circles. The solidus and liquidus points are represented in blue and red, respectively) and the ideal behavior (dotted line) determined with the Schröder-van Laar relation are shown.

In order to apprehend the formation of the equimolar compound for Lido:dlM and verify whether other stoichiometric cocrystals could also be formed, phase diagrams between dl-menthol and Lido:dlM cocrystal on the one hand, and between Lido:dlM cocrystal and lidocaine on the other hand, have been established from the differential scanning calorimetry (DSC) analysis of the related mixtures (Figure 5).

Here, a unique 1:1 stoichiometric compound with a congruent melting point has been found from the stable phase diagram of the lidocaine/dl-menthol system (Figure 5B), following the same tendency as the lidocaine/l-menthol system already published [44]. However, excepting the existence of the equimolar cocrystal, the binary phase diagram obtained with the racemic menthol differs from that of the enantiopure terpene-based binary phase diagram. Firstly, in the dl-menthol-rich region of the new phase diagram, the eutectic point is slightly shifted towards high lidocaine content and the temperature of invariant equilibrium decreases by almost 7 °C (Table 4) compared to the enantiopure menthol-based phase diagram.

Interestingly, this 7 °C variation corresponds to the difference in melting point between Lido:lM and Lido:dlM as discussed in Section 3.1. As far as the lidocaine-rich region of the lidocaine/dl-menthol stable phase diagram is concerned, the eutectic point is close to the cocrystal melting point (Table 4), indicating that only one liquidus curve can be monitored, i.e., the lidocaine one. This implies that, at the invariant equilibrium of this region in the phase diagram, the cocrystal content of the physical mixture between Lido:dlM and lidocaine melts completely at any lidocaine molar ratio. This finding can be explained by the high melting temperature difference between lidocaine and Lido:dlM (ΔT ~ 37 °C). In contrast, for the Lido:lM/lidocaine physical mixture melting, complete melting of the cocrystal at the invariant equilibrium was observed for lidocaine molar ratios higher than 0.61 (ΔT_fus, lidocaine vs. l-menthol_ ~ 29 °C). In this region of the phase diagram, the temperature of the invariant equilibrium is around 6 °C lower compared to that of the lidocaine/l-menthol stable phase diagram. Surprisingly, the solidus and liquidus experimental points (Figure 5B, blue and red points, respectively) fit the ideal behavior of the system calculated from the Schröder—van Laar equation [44,65,66,67] (Figure 5B, dotted lines), suggesting an ideal behavior in the lidocaine/dl-menthol molten state, i.e., with no supplementary intermolecular interactions compared to the related solid state of the physical mixture. Indeed, the temperatures of the two experimental invariant equilibria are exactly the same as that of the two ideal behaviors, respectively. Consequently, the excess in Gibbs energy of the liquid phase at the eutectic point is null for both regions of the lidocaine/dl-menthol stable phase diagram, while it has been previously found to be −430 ± 27 and −600 ± 38 J·mol^−1^ for the l-menthol and lidocaine rich regions, respectively [44]. These results can also be apprehended by visualizing and comparing the deviation of the experimental data from ideality for each region of the menthol racemate-based phase diagram on the one hand, and the enantiopure menthol-based one on the other hand.

One can conclude from the analysis and comparison of the menthol racemate- and enantiopure menthol-based binary phase diagrams with lidocaine that replacing Lido:lM with Lido:dlM (i) reduces the congruent melting region of the cocrystal, (ii) decreases the two distinguishable temperatures of the invariant equilibria with the same amplitude as the melting point difference, and (iii) breaks down the supplementary intermolecular interactions in the molten state compared to the solid state regardless of lidocaine molar content. These findings will help understand further differences in the properties of the racemic and the enantiopure cocrystals.

### 3.4. Screening of the Solid–Liquid and Gas–Liquid Equilibria for the Racemic and Enantiopure Cocrystals

**Figure 6:**
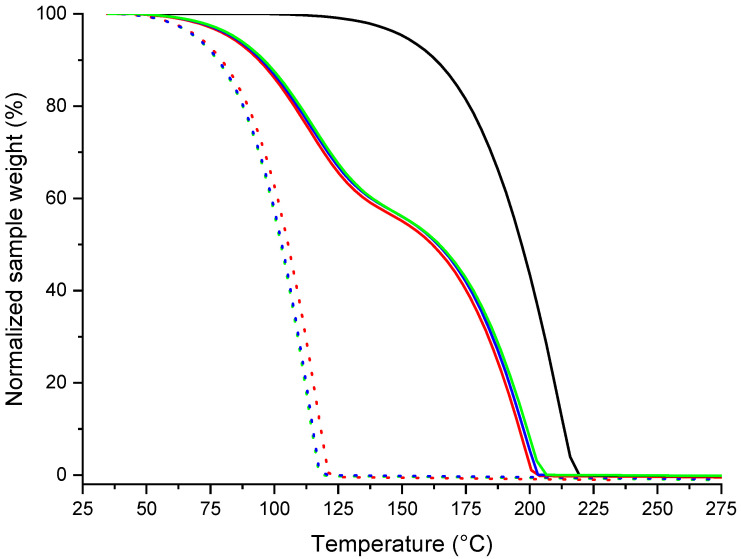
Thermogravimetric curves obtained for the Lido:dlM (red solid line), Lido:lM (green solid line), and Lido:dM (blue solid line) cocrystals. For comparison, the data were also collected for pure lidocaine (black solid line), racemic (red dotted line), levo- (green dotted line), and dextro- menthol (blue dotted line). Uncertainties: Δx = 0.6 °C, and Δy = 0.4%.

Menthol and lidocaine stability within the cocrystals were established by thermogravimetric analysis (TGA). Taken separately after melting, racemic and enantiopure menthol evaporate at 55 °C, and lidocaine evaporates at 125 °C (Figure 6). The thermal gravimetric behavior of the three cocrystals is interposed between that of menthol entities and lidocaine, indicating specific interactions in the molten state of the cocrystal. In that case, menthol and lidocaine evaporations are skewed towards lower and higher temperatures, respectively. However, the TGA curve of the Lido:dlM cocrystal is slightly shifted towards lower temperatures compared to the TGA curves of the two cocrystals prepared with the enantiopure menthol entities. This result agrees with the fact that the menthol racemate-based physical mixtures present no excess energy in their molten state contrary to the enantiopure menthol-based ones. Furthermore, interestingly, these results confirm that the lidocaine/menthol association remains in the molten state until ~126 °C with a rich fraction of menthol, and until ~203 °C with a rich fraction of lidocaine, (i) confirming the formation of the cocrystals by crystallization of the molten state; and (ii) suggesting the potential formation of the cocrystals by condensation of the gaseous phase, in these related temperature ranges. Furthermore, the TGA experiments confirmed the stability of both Lido:lM and Lido:dlM cocrystals at room temperature. The latter result was also confirmed by complementary experiments regarding the behavior of the cocrystals after exposition with air at room temperature, ca. 20 °C (Table S3). Indeed, after more than one week of exposition, no change in the sample weight or the thermal analysis curves was noticed. Interestingly, the powder and single crystal of each cocrystal can be stored in a sealed flask for at least 10 years without any chemical or physical modification (*cf*. Section 2 Materials and Methods). All these stability data prove that, although the Lido:dlM cocrystal is less stable than the enantiopure menthol-based one, no resolution occurs when the former is placed at atmospheric pressure, at a temperature lower than its melting point.

**Figure 7:**
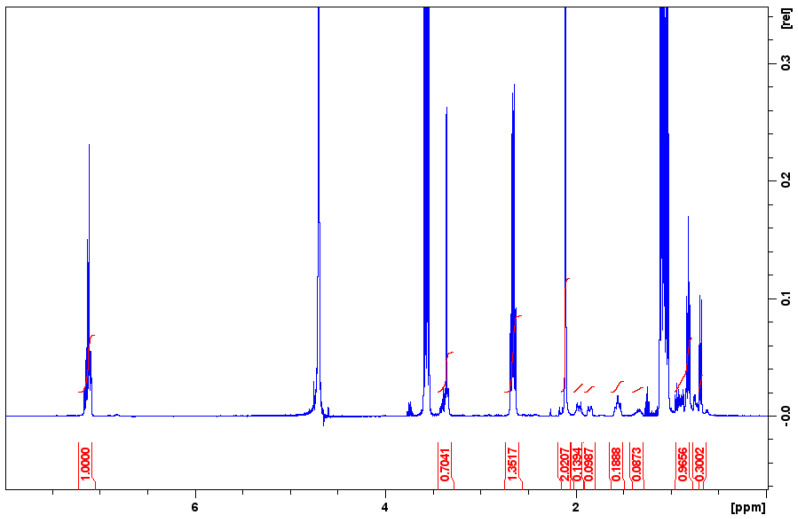
^1^H NMR spectrum of the lidocaine/dl-menthol system after solubilization of the Lido:dlM cocrystal in D_2_O for 24 h. x axis: chemical shift δ in ppm (part per million), y axis: integral (number of proton) in relative unit. The red curves correspond to the integration peak: The height of the curve is proportional to the number of protons.

The influence of racemic menthol on the lidocaine/menthol cocrystal characteristic has been evaluated by ^1^H NMR spectroscopy in D_2_O (Figure 7). Hence, the suitability of recovering lidocaine and dl-menthol NMR signals without any shift of their related signals has been proven (*cf.* Figure S2). The latter supports the fact that Lido:dlM cocrystal does not present supplementary interactions in the molten state, and thus, by interpolation, in the liquid state. The same results were obtained in a non-polar solvent, namely CDCl_3_ (*cf.* Figure S3). Furthermore, the same dipolar interactions were detected for the Lido:dlM and the lidocaine/dl-menthol mixture with the same molar ratio by comparing their respective 2D NOESY spectrum (*cf.* Figure S4).

From these data, e.g., lower melting point, lower heat of fusion, and no excess in Gibbs energy in the liquid state for the menthol racemate-based compared to the enantiopure menthol-based cocrystal, one can wonder if the solubility of Lido:dlM cocrystal is impacted due to the presence of the racemic menthol in the cocrystal formulation. The following section will deal with the unprecedented dissolution survey of both racemic and enantiopure menthol-based cocrystals.

### 3.5. Dissolution Behavior Assessment and Comparison of the Racemic and Enantiopure Cocrystals

**Figure 8:**
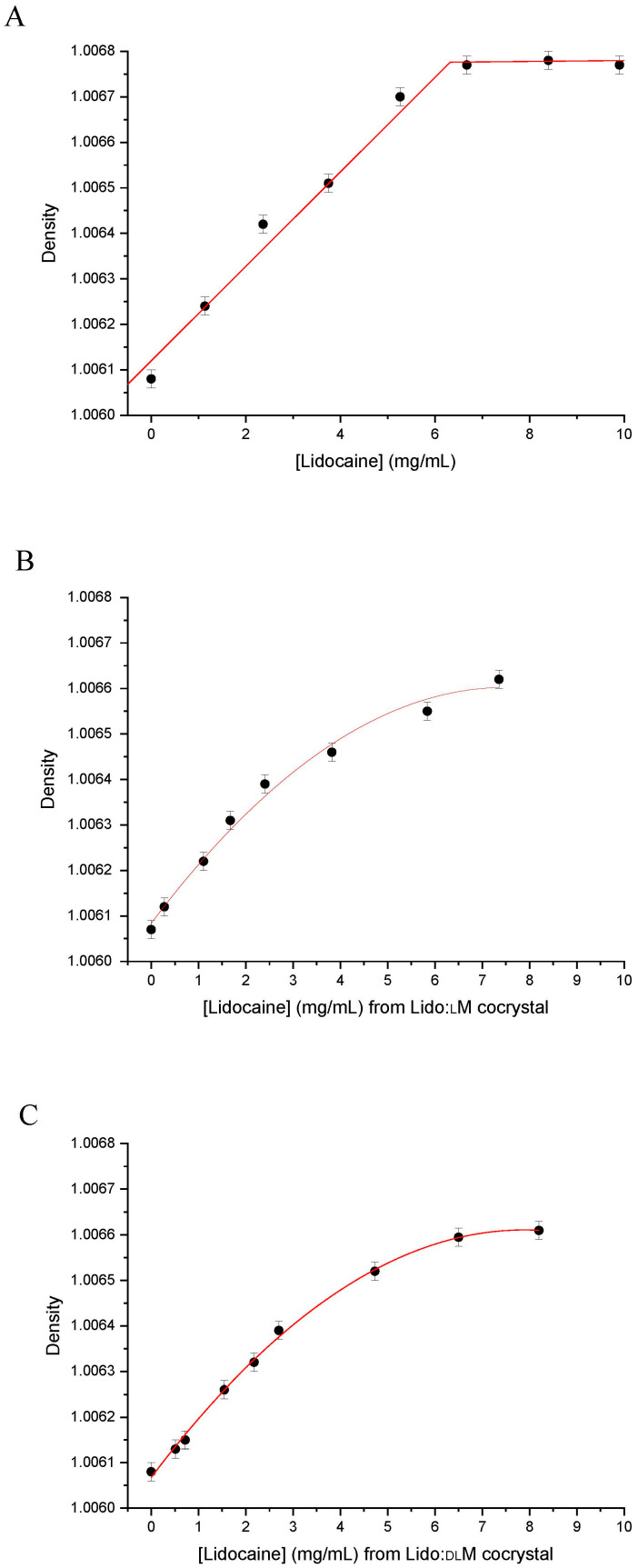
Density measurement as a function of the lidocaine content in lidocaine (**A**); Lido:lM cocrystal (**B**); and Lido:dlM cocrystal (**C**) solubilized in phosphate buffer.

Considering that the density of a solution is proportional to the concentration of the solute in the selected solvent, the high accurate density measurement technique has been chosen for evaluating the saturation concentration of both Lido:dlM and Lido:lM cocrystals, compared to pure lidocaine data in the same environmental conditions, i.e., pH = 7.2, temperature = 25 °C. (Figure 8). In the latter conditions, pure lidocaine presents a saturation concentration of 6.3 mg·mL^−1^, while 6.9 and 9.1 mg of lidocaine per mL of buffer solution can be dissolved at the highest extrapolated amount from Lido:lM (11.5 mg·mL^−1^) and Lido:dlM (15.2 mg·mL^−1^), respectively. This suggests that the solubility of lidocaine from Lido:dlM cocrystal is enhanced compared to its solubility from Lido:lM cocrystal, which is slightly improved compared to pure lidocaine.

These results were also confirmed by the determination of the slope of the density evolution as a function of the lidocaine content presented in Figure 8. The data, gathered in Table 5, indicate an improvement in the racemic cocrystal solubility. This is in agreement with the low stability of the racemic cocrystal compared to the enantiopure one. It is worth noting here that in the range of the concentration tested, both cocrystals do not exhibit a clear solubility plateau compared to pure lidocaine. This can be explained by a specific equilibrium of dissolution that will be apprehended by visualizing the dissolution process.

Hence, in order to obtain deeper insights into the biopharmaceutical properties of both cocrystals, the dissolution kinetics were evaluated in static conditions in the same buffer solution used for the density experiments, at 25 °C, i.e., at a temperature much lower than the Lido:dlM cocrystal melting point. The results are gathered in Table 6. Firstly, it can be deduced that the dissolution kinetics of the Lido:dlM cocrystal is higher than that of the Lido:lM, both being higher than the corresponding ones for lidocaine or menthol separately. This claim confirms the previous results related to the solubility of the two cocrystals. Moreover, the optical images of Table 6 allow us to propose that the dissolution of each cocrystal is achieved through (i) the instantaneous formation of a lidocaine/menthol organic oily phase from the solid cocrystal followed by (ii) the dissolution of the oily phase in the buffer aqueous solution, mostly for the racemic cocrystal. This unexpected melting/dissolution behavior of the Lido:dlM can be explained by (i) the lower melting point of the cocrystal (only 7 °C higher than the working temperature for the dissolution studies), and (ii) no supplementary intermolecular interactions in Lido:dlM molten state noticed compared to its solid state. As far as the Lido:lM cocrystal is concerned, its 7 °C higher melting point than the working temperature for the dissolution studies, and the slight interactions in the molten state [44] have an impact on the lower kinetics of dissolution compared to the menthol racemate-based cocrystal. No complete oily phase formation from the cocrystal has been observed (Table 6). The lidocaine/menthol/water tertiary phase diagram will be investigated in future work to obtain more insights into the liquid–liquid phase separation kinetics highlighted in this study.

Nevertheless, it is worth noting that at high concentrations, the cocrystal solubility was underestimated since the oily phase was stuck to the vial walls. This could explain the asymptote for the curve representing density vs. concentration for both cocrystals (Figure 8). Consequently, further experiments for determining the dissolution kinetics were performed with a higher amount of Lido:dlM than the estimated solubility of the cocrystal (Table 7). Interestingly, after 4 min of immersion in the buffer solution, the entire cocrystal powder was in the molten/liquid state. In these conditions, two interpretations can be proposed: (i) the oily phase is not totally dissolved in the aqueous medium, and (ii) a supersaturated solution is formed. This implies that the cocrystals of this study may have a higher global solubility than estimated.

This thorough characterization of the enantiopure and the menthol racemate-based cocrystals demonstrates the feasibility of altering the biopharmaceutical properties of a given stoichiometric compound [68] by replacing the enantiopure constituent of the cocrystal with the related racemate. The latter is mostly due to the disordered crystal structure of the racemic cocrystal compared to the enantiopure one.

## 4. Conclusions

Racemic forms are of common use in the pharmaceutical field to decrease the cost of the final products. Our analysis of the lidocaine/dl-menthol binary system showed really promising results such as a decrease in the melting point of the related 1:1 stoichiometric compound and an enhancement of its solubility in comparison to the lidocaine/levomenthol cocrystal. The results were presented after running tests in our previous discovery and the new dl-menthol/lidocaine utilizing single crystal X-ray, XRPD, FTIR, DSC, Raman, thermal degradation (TGA), NMR, and solubility evaluation. For the first time, the FTIR, Raman, and dissolution studies of the cocrystals were presented for this new and previous cocrystal providing even further details. All the results gathered were used to attribute a physico-chemical hierarchy of stability for the enantiopure and menthol racemate-based cocrystals, showing promising properties in the pharmaceutical field by developing new formulation strategies for lidocaine. Furthermore, the results obtained can complete a survey concerning the influence of racemization and enantiopurity on cocrystal formation [21]. With that in mind, our next step will evaluate the pharmacokinetics of lidocaine after cocrystal administration and estimate the cytotoxicity of the new formulations. Overall, the results of the present study may suggest pharmaceutical preparation of the lidocaine/racemic menthol cocrystal in its oily phase dispersed in water via emulsion formulation for topical anesthesia (e.g., endoscopy, transdermal application) or as sublingual tablets for local anesthesia of the oropharyngeal sphere (e.g., buccal ulcerations, sore throat). The screening strategy that we propose here can be applied to other cocrystals in order to obtain more insights into cocrystals’ design [69].

## Figures and Tables

**Table 1:** Crystal data and structure refinement for the Lido:dlM cocrystal recorded at 233 K (Cambridge Crystallographic Data Centre reference number: 2248363).

Formula		C_24_H_42_N_2_O_2_
FW (g·mol^−1^)		390.59
Temperature		233 K
Wavelength		1.54184 Å
Cryst. Syst.		Monoclinic
Space group		P 2_1_/c
Unit cell Dimensions	a (Å) b (Å) c (Å)	8.4947(3) 22.2025(6) 13.4443(3)
	α (°) β (°) γ (°)	90 93.162(3) 90
Volume (Å^3^)		2531.78(13)
Z		4
D_x_ (g·cm^−3^)		1.025
µ (mm^−1^)		0.496
Final R_1_ [I > 2σ(I)]		0.1053
wR_2_ (all data)		0.1971
S		1.038

**Table 2:** Thermal characteristics of the 1:1 lidocaine:dl-menthol cocrystal compared with the characteristics of lidocaine, racemic menthol, enantiopure menthol, and l-menthol-based cocrystal.

	Lido:dlM	dl-Menthol	Lidocaine	Lido:lM	l-Menthol
T_fus_ (°C)	32.0 ± 0.3	33.8 ± 0.3	68.6 ± 0.5	39.1 ± 0.2	42.9 ± 0.3
Δ_fus_H (kJ per mol of pure component)	28.6 ± 0.3	14.2 ± 0.2	16.9 ± 0.2	38.3 ± 0.3	14.1 ± 0.2
Ref.	This work	[37]	[44]	[44]	[44]

**Table 3:** Raman spectroscopic results obtained for solid enantiopure and the menthol racemate-based cocrystals, and the components taken separately. υ: stretching, δ: in-plane bending, γ: out-of-plane bending, τ-torsion, and ω- wag vibrations. Data in bold indicate main signals. Menthol-exclusive bands are underlined.

Signal Attribution	Wavenumber (cm^−1^)				
	Lidocaine	l-Menthol	dl-Menthol	Lido:lM	Lido:dlM
υ_NH_				3226	3232
υ_CH_	3043			3045	3043
υ_CH_	2967	**2964**	**2958**	2953	2955
υ_CH_	**2921**	**2931**	**2926**	2932	2923
υ_CH_	2874	**2859**	**2867**	2856	2872
υ_CH_	2722			2722	2725
υ_C=O_	**1660**			**1660**	**1661**
δ_HNC_				**1652**	**1651**
υ_CN_, δ_HNC_	**1591**			1590	1592
δ_CH_	1450	1455/1445	1455/1443	1447	1450
δ_CH_	1377/1371			1377/1361	**1374/1361**
δ_OH_		1345	1345	1343	1343
δ_CH_		1304	1307	1303	1307
υ_ring_	**1261**			1264	1261
υ_CO_		1240	1240	1239	1240
υ_CN_	1208			1208	**1210**
υ_CN_	1161			1161	1163
δ_CH_	**1092**			1090	**1093**
ν_CC_, δ_NCO_	**989**			990	989
υ_CC_		966/954	965/952	966/956	967/955
υ_CC_		919	919	918	920
δ_ring_, υ_CC_	875	**876**	877	**873**	875
γ_CH_		**809**	809	**803**	808
γ_CH_		768	**767**	767	766
ω_HNC_. δ_ring_	**752**			752	752
δ_ring_	**703**			704	702
ω_NCO_	**616**			616	614
τ_NCO_, δ_ring_		**546/540**	**553**	**543**	**546**
τ_ring_		499	500	**508/501**	**509/501**
δ_ring_, δ_CH2_	487			470	488
ω_NC2_	402	**404**	**408**	404	405
δ_CC_					**324**
Lattice vib.		**291**	**289**	293	287
Lattice vib.		**264**	**258**		258
ω_CC_	227			230	**229**

**Table 4:** Temperature and composition data of the eutectic points for the lidocaine/dl-menthol phase diagram obtained in the present study and compared with the data of the lidocaine/l-menthol phase diagram reported in reference [44]. The eutectic points for the lidocaine/dl-menthol system were determined with the Tammann plots described in reference [64] (*cf.* Figure S5).

Lidocaine/dl-Menthol		Lidocaine/l-Menthol	
*x*_e_	T_e_ (°C)	*x*_e_	T_e_ (°C)
0.215 ± 0.003	21.7 ± 0.3	0.20	28.6
∈ [0.50, 0.57]	32.0 ± 0.3	0.61	37.6

**Table 5:** Data calculated from the density = f(concentration) curves at 25 °C and pH 7.2. The solubility of each menthol compound conducted to aqueous concentrations lower than the limit of detection of the density device. For each cocrystal, the slope of the d = f(C) straight line was determined in the 0–3 mg·mL^−1^ concentration range.

	Lidocaine	Lido:lM	Lido:dlM
Lidocaine solubility (mg·mL^−1^)	6.3 ± 0.1	–	–
Cocrystal estimated solubility (mg·mL^−1^)	–	11.5	15.2
Lidocaine solubility from the cocrystal (mg·mL^−1^)	–	6.9 ± 0.1	9.1 ± 0.1
Slope of the d = f(C) curve (×10^4^ mL·mg^−1^)	1.04 ± 0.02	1.05 ± 0.02	1.16 ± 0.01

**Table 6:** Images illustrating the kinetics of dissolution of the lidocaine:l-menthol and lidocaine:dl-menthol cocrystals (7.2 mg·mL^−1^) compared with the pure constituents, i.e., lidocaine (4.3 mg·mL^−1^), and dl-menthol (2.9 mg·mL^−1^) at 25 °C. Width of each image: 4 cm.

		Lido:dlM	Lido:lM	Lidocaine	dl-Menthol
	Anhydrous medium	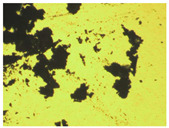	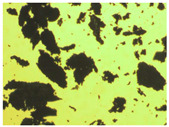	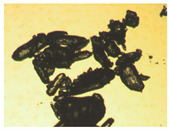	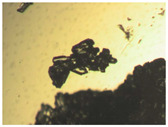
Time after hydration (min)					
0		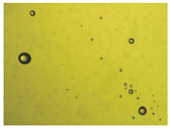	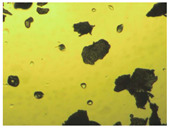	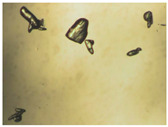	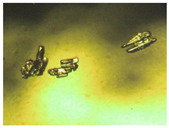
10		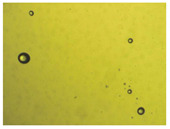	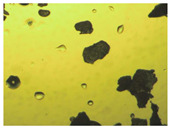	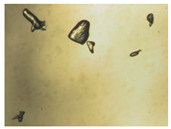	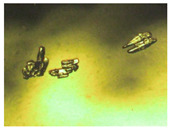
50		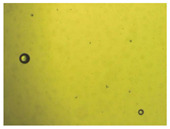	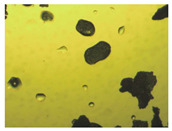	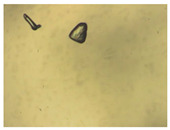	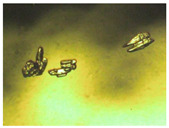
380		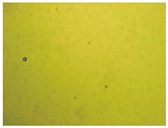	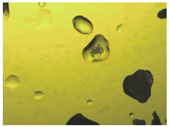	–	–
560		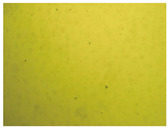	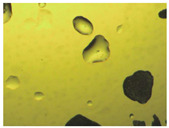	–	–

**Table 7:** Images illustrating the kinetics of dissolution of the lidocaine:dl-menthol cocrystal (17 mg·mL^−1^) compared with pure constituents, i.e., lidocaine (10.2 mg·mL^−1^) and dl-menthol (6.8 mg·mL^−1^) at 25 °C. Width of each image: 4 cm.

		Lido:dlM	Lidocaine	dl-menthol
	Anhydrous medium	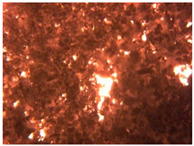	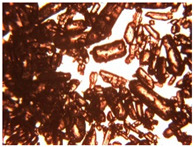	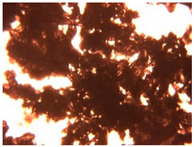
Time after hydration (min)				
0		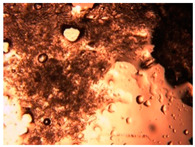	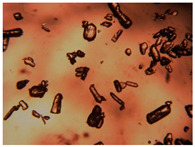	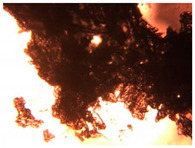
4		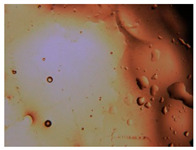	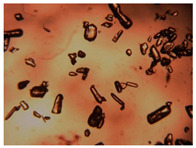	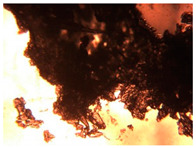
60		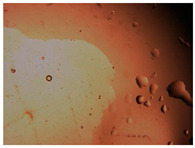	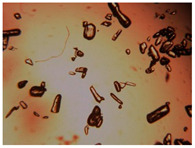	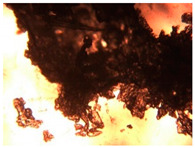
268		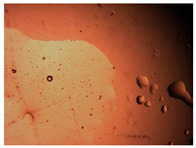	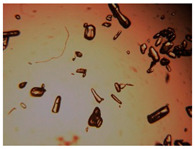	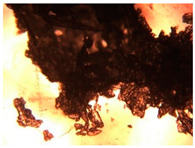
569		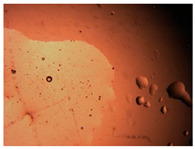	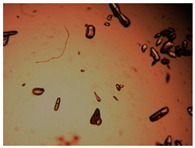	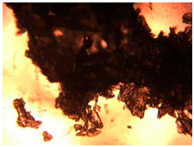

## Data Availability

Not applicable.

## Supplementary Materials

The following are available online at https://www.mdpi.com/article/10.3390/pharmaceutics15041102/s1, Table S1: Torsion angle data for the Lido:LM and Lido:DLM cocrystals; Table S2: Hydrogen bond geometry in the Lido:LM (data in bold) and Lido:DLM cocrystals; Figure S1: Raman spectra obtained in the 3500–2400 cm^−1^ (A) and 1700–200 cm^−1^ wavenumbers range (B); Figure S2: ^1^H NMR spectrum of pure lidocaine (A) and menthol (B) dissolved in D_2_O; Figure S3: ^1^H NMR spectrum of pure lidocaine (A) and dl-menthol (B), as well as the Lido:dlM cocrystal (C) dissolved in CDCl_3_; Figure S4: ^1^H NMR 2D NOESY spectrum of Lido:dlM cocrystal (A) and the mixture of lidocaine and dl-menthol at the same molar ratio (B) dissolved in CDCl_3_; Figure S5: Tammann plots established from the eutectic equilibrium data of the stable lidocaine/dl-menthol phase diagram; Table S3: Melting point and heat of fusion of the Lido:dlM and Lido:lM cocrystals as a function of the time of exposition in air at room temperature, ca. 20 °C.
