# Supplementary material for: New Lidocaine-Based Pharmaceutical Cocrystals: Preparation, Characterization, and Influence of the Racemic vs. Enantiopure Coformer on the Physico-Chemical Properties
Source: Pharmaceutics. 2023 Mar 29;15(4):1102. doi: 10.3390/pharmaceutics15041102 (PMC10142540; doi:10.3390/pharmaceutics15041102)
Supplement: Supplementary file 1 [file pharmaceutics-15-01102-s001.zip › pharmaceutics-2161170-supplementary.pdf]

New Lidocaine-Based Pharmaceutical Cocrystals: Preparation, Characterization and Influence of the Racemic *versus* Enantiopure Coformer on the Physico-chemical Properties

Panpan Ma[†1], Balthazar Toussaint[†1,2] Enrica Angela Roberti[1], Noémie Scornet[1], Axel Santos Silva[1], Luis Castillo Henríquez[1], Monique Cadasse[3], Philippe Négrier[4], Stéphane Massip[5], Hanh Dufat[6], Karim Hammad[6], Cecilia Baraldi[7], Maria Cristina Gamberini[7], Cyrille Richard[1], Stéphane Veesler[8], Philippe Espeau[1], Tu Lee[9], and Yohann Corvis[1,*]

[1]*Université Paris Cité, CNRS, INSERM, UTCBS, Chemical and Biological Technologies for Health Group (utcbs.u-paris.fr), F-75006 Paris, France.*

[2]*Département recherche et développement pharmaceutique (DRDP), Agence générale des équipements et produits de santé, AP-HP, F-75005 Paris, France.*

[3]*Université Paris Cité, Département Physico-Chimie du Médicament, Unité Pédagogique de Chimie Analytique, Physique et toxicologie, Faculté de Santé, 4 Avenue de l'Observatoire, F-75006 Paris, France.*

[4]*Université de Bordeaux, Laboratoire Ondes et Matière d'Aquitaine, UMR 5798, F-33400 Talence, France.*

[5]*Université de Bordeaux, CNRS, INSERM, IECB, UAR 3033, F-33600 Pessac, France.*

[6]*Université Paris Cité, CiTCoM, UMR CNRS 8038, F-75006 Paris, France.*

[7]*Department of Life Sciences, University of Modena and Reggio Emilia, Modena, Cap 41125, Italy.*

[8]*CNRS, Aix-Marseille Université, CINaM (Centre Interdisciplinaire de Nanosciences de Marseille), Campus de Luminy, F-13288, Marseille cedex 09, France.*

[9]*Department of Chemical and Materials Engineering, National Central University, Taoyuan City 320317, Taiwan (R.O.C.).*

[†]Both authors contributed equally to this research article.

*Author to whom correspondence should be addressed.

Table S1. Torsion angle data for the Lido:LM and Lido:DLM cocrystals

	Lido:LM cocrystal		Lido:DLM cocrystal			
	Atoms	Angle (deg.)	Atoms	Angle (deg.)	Atoms	Angle (deg.)
Lidocaine	C31-N32-C35-C36	-71.5(3)	C12-N13-C14-C15	-68.2(5)		
	C31-N32-C33-C34	66.6(3)	C12-N13-C16-C17	68.7(4)		
	C29-N28-C25-C20	103.0(2)	C10-N9-C6-C1	95.2(4)		
	C29-N28-C25-C24	-76.1(3)	C10-N9-C6-C5	-85.6(4)		
			Disordered menthol (I)		Disordered menthol (II)	
L-menthol	C5-C6-C9-C10	-62.3(2)	C25-C26-C29-C30	-78.6(4)	C45-C46-C49-C50	-79.1(5)
	C5-C6-C9-C11	62.9(3)	C25-C26-C29-C31	42.2(4)	C45-C46-C49-C51	46.1(5)
	C1-C6-C9-C10	172.75(18)	C21-C26-C29-C30	152.0(3)	C41-C46-C49-C50	153.1(4)
	C1-C6-C9-C11	-62.1(3)	C21-C26-C29-C31	-87.1(4)	C41-C46-C49-C51	-81.7(4)

Table S2. Hydrogen bond geometry in the Lido:LM (data in bold) and Lido:DLM cocrystals

			Length (Å)			Angle (deg.)
H-bond type	D−H···A	Menthol	D−H	H···A	D···A	D−H···A
intermolecular	N−H···O		**0.870**	**2.213**	**3.028(2)**	**156**
		(I)	0.870	2.240	2.997(4)	145
		(II)	0.870	2.060	2.836(5)	148
	O−H···O		**0.830**	**2.084**	**2.817(2)**	**147**
		(I)	0.830	2.060	2.749(4)	140
		(II)	0.830	1.940	2.754(5)	167
intramolecular	N−H···N		**0.870**	**2.309**	**2.731(2)**	**110**
			0.870	2.300	2.721(3)	110

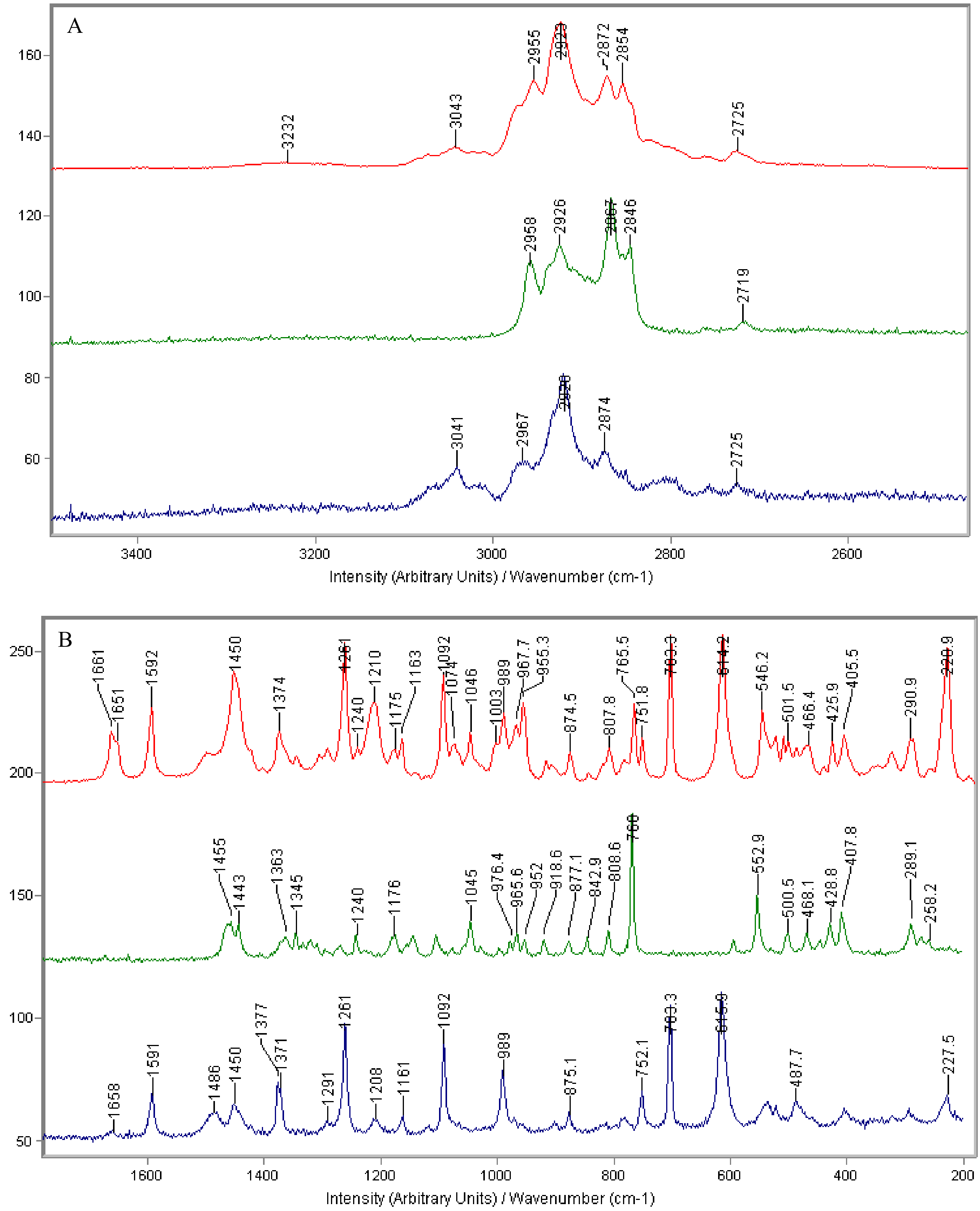

Figure S1. Raman spectra obtained in the 3500-2400 cm^{-1} (A) and 1700-200 cm^{-1} wavenumbers range (B). Color code: (Red) Lido:DLM cocrystal, (green) pure DL-menthol, and (blue) lidocaine.

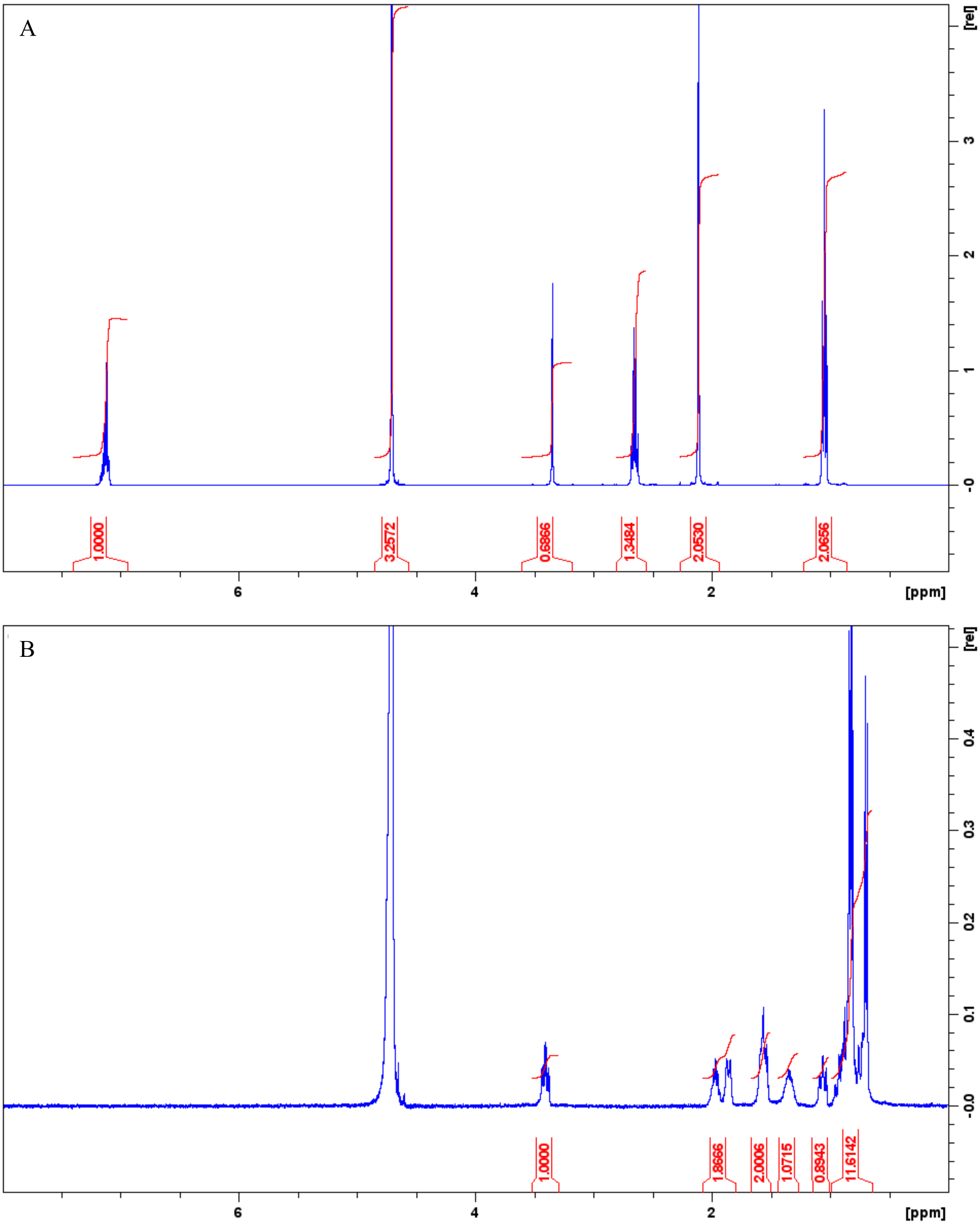

Figure S2. ^{1}H NMR spectrum of pure lidocaine (A) and menthol (B) dissolved in D_2O.

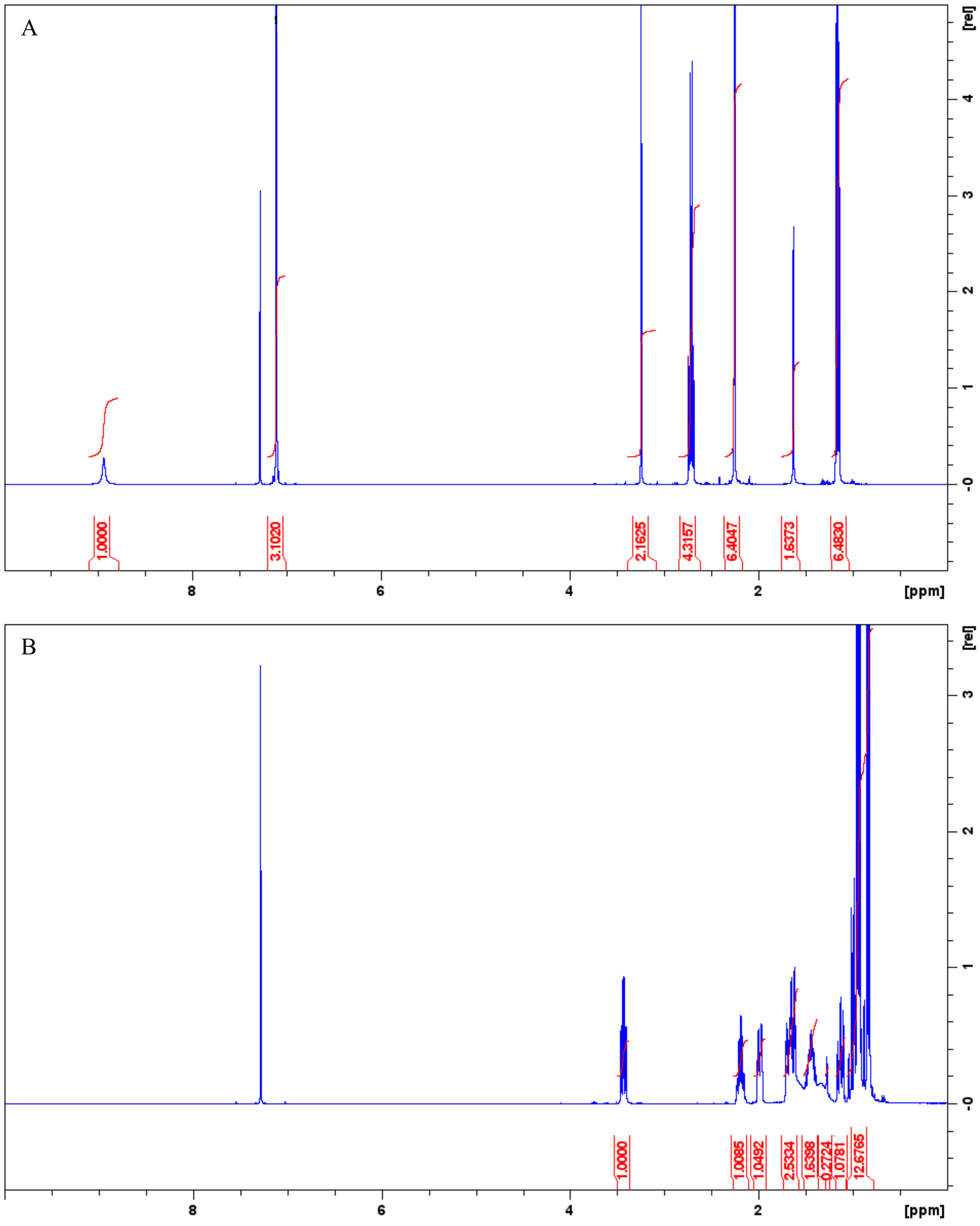

A
1.0000
3.1020
2.1625
4.3157
6.4047
1.6373
6.4830
[rel]
[ppm]
B
1.0000
1.0085
1.0492
2.5334
1.6398
0.2724
1.0781
12.6765
[rel]
[ppm]

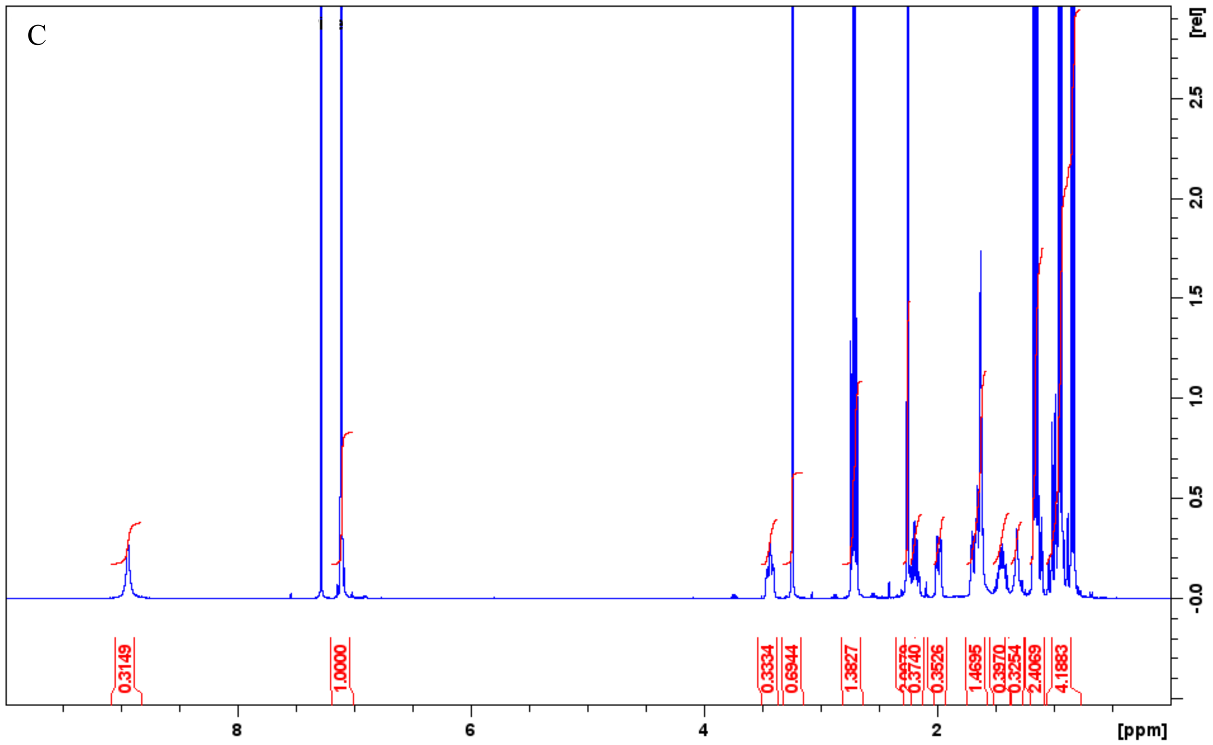

Figure S3. ^{1}H NMR spectrum of pure lidocaine (A) and DL-menthol (B), as well as the Lido:DLM cocrystal (C) dissolved in $CDCl_3$.

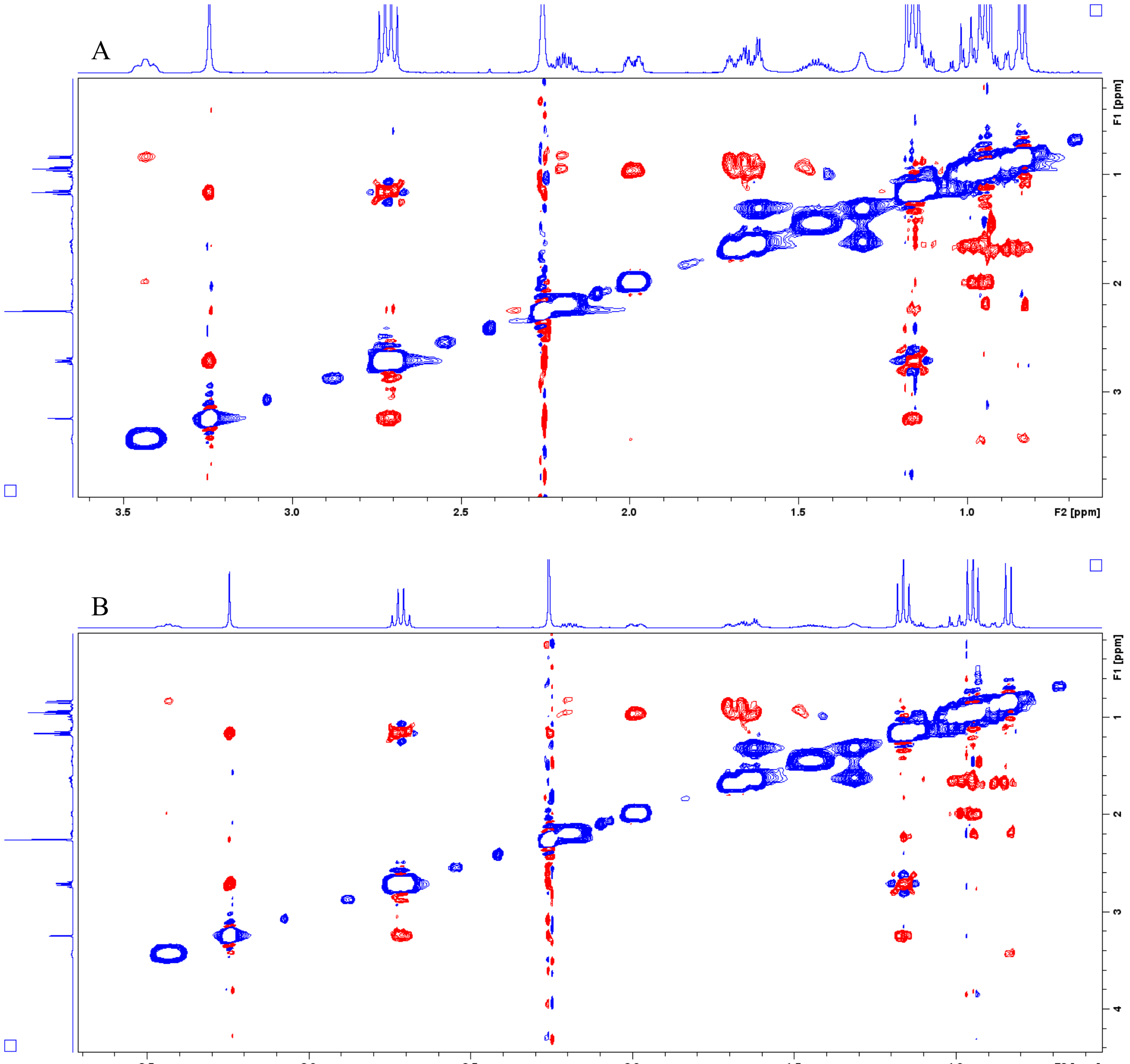

Figure S4. ^{1}H NMR 2D NOESY spectrum of Lido:DLM cocrystal (A) and the mixture of lidocaine and DL-menthol at the same molar ratio (B) dissolved in $CDCl_3$.

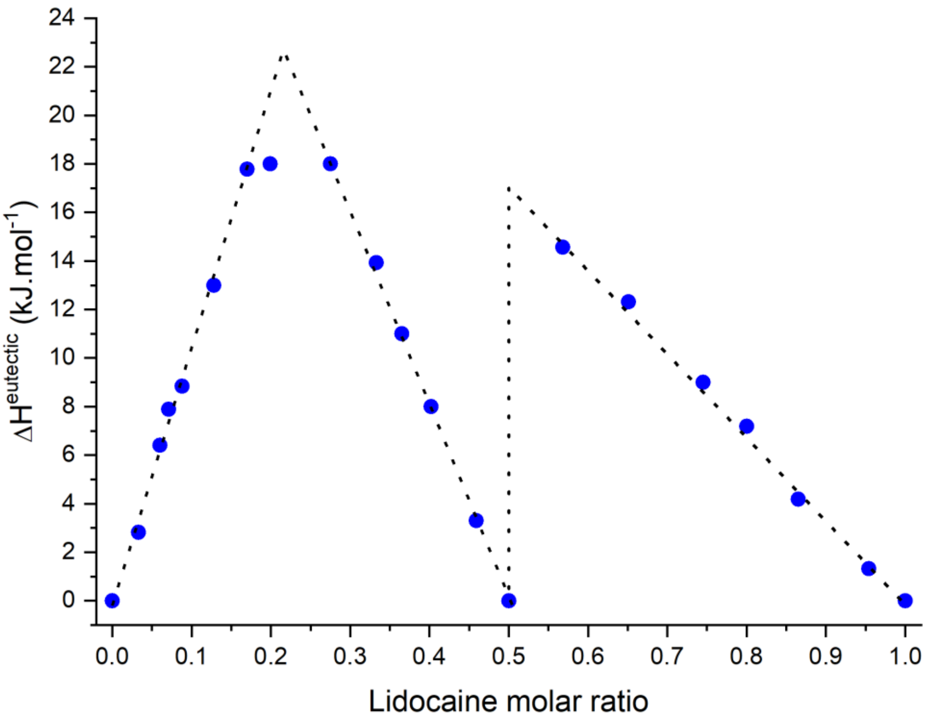

Figure S5. Tammann plots established from the eutectic equilibrium data of the stable lidocaine/DL-menthol phase diagram.

Table S3. Melting point and heat of fusion of the Lido:DLM and Lido:LM cocrystals as a function of the time of exposition in air at room temperature, *ca.* 20 °C

Annealing time at rt (h)	Lido:DLM		Lido:LM	
	T_{fus} (°C)	$\Delta_{fus}H$ (kJ per mol of pure component)	T_{fus} (°C)	$\Delta_{fus}H$ (kJ per mol of pure component)
0	38.7	37.7	32.0	29.0
63	39.0	38.0	32.7	29.1
147	38.9	37.9	32.7	29.3
201	39.1	38.0	32.4	29.1
Mean value	38.9	37.9	32.5	29.1
Standard deviation	0.2	0.1	0.3	0.1
